# Potentially zoonotic pathogens and parasites in opportunistically sourced urban brown rats (*Rattus norvegicus*) in and around Helsinki, Finland, 2018 to 2023

**DOI:** 10.2807/1560-7917.ES.2024.29.40.2400031

**Published:** 2024-10-03

**Authors:** Tuomas Aivelo, Hussein Alburkat, Nina Suomalainen, Rebekka Kukowski, Petra Heikkinen, Antti Oksanen, Otso Huitu, Rauni Kivistö, Tarja Sironen

**Affiliations:** 1 Science Communication & Society, Institute of Biology, University of Leiden, Leiden, The Netherlands; 2 Organismal and Evolutionary Biology research program, Faculty of Biological and Environmental Sciences, University of Helsinki, Helsinki, Finland; 3 Department of Virology, Faculty of Medicine, University of Helsinki, Helsinki, Finland; 4 Finnish Food Authority, Animal Health Diagnostic Unit (FINPAR), Oulu, Finland; 5 Natural Resources Institute Finland, Helsinki, Finland; 6 Department of Food Hygiene and Environmental Health, Faculty of Veterinary Medicine, University of Helsinki, Helsinki, Finland; 7 Department of Veterinary Biosciences, Faculty of Veterinary Medicine, University of Helsinki, Helsinki, Finland

**Keywords:** synanthropic animals, rodent-borne zoonosis, urban wildlife, Northern Europe, pest management

## Abstract

**Background:**

Brown rats (*Rattus norvegicus*) are synanthropic rodents with worldwide distribution, which are known to harbour many zoonotic pathogens and parasites. No systematic zoonotic surveys targeting multiple pathogens and parasites have previously been conducted in urban rats in Finland.

**Aim:**

In Helsinki, Finland, we explored the presence and prevalence in brown rats of certain pathogens and parasites (including helminths, viruses and bacteria) across potentially zoonotic taxa.

**Methods:**

We opportunistically received rat carcasses from pest management operators and citizens from 2018 to 2023. We searched for heart- or lungworms, performed rat diaphragm digestion to check for *Trichinella* and morphologically identified intestinal helminths. We assessed virus exposure by immunofluorescence assay or PCR, and detected bacteria by PCR (*Leptospira*) or culture (*Campylobacter*).

**Results:**

Among the rats investigated for helminths, no heart- or lungworms or *Trichinella* species were detected and the most common finding was the cestode *Hymenolepis nana* (in 9.7% of individuals sampled, 28/288). For some of the surveyed virus taxa, several rats were seropositive (orthopoxviruses, 5.2%, 11/211; arenaviruses, 2.8%, 6/211; hantaviruses 5.2%, 11/211) or tested positive by PCR (rat hepatitis E virus, 1.8%, 4/216). *Campylobacter jejuni* (6.6%, 17/259) and *Leptospira interrogans* (1.2%, 2/163) bacteria were also present in the rat population examined.

**Conclusions:**

Prevalences of potentially zoonotic pathogens and parasites in brown rats in Helsinki appeared low. This may explain low or non-existent diagnosis levels of rat-borne pathogen and parasite infections reported in people there. Nevertheless, further assessment of under-diagnosis, which cannot be excluded, would enhance understanding the risks of zoonoses.

**Key public health message**

**What did you want to address in this study and why?**

Brown rats have been shown to host several zoonotic pathogens and parasites and they are believed to be important sources of human infections in various environmental and social contexts. We aimed to survey for the presence of several important potentially zoonotic parasites and pathogens in brown rats in Helsinki, Finland, from 2018 to 2023. These included worms (e.g. *Trichinella and Hymenolepis nana*), viruses, and bacteria (e.g. *Campylobacter jejuni* and *Leptospira interrogans*).

**What have we learnt from this study?**

Whereas we did not detect *Trichinella* and heart- and lung worms in the rats examined, we observed most of the zoonotic pathogens and parasites that we searched for. These included *H. nana* (9.7% of rats investigated), rat hepatitis E virus (1.8% of rats surveyed), as well as *C. jejuni* and *L. interrogans* (6.6% and 1.2% of rats surveyed respectively). Nevertheless, many of the prevalences in our study seemed lower than in other European cities and the reasons for this remain unexplored.

**What are the implications of your findings for public health?**

We found a low prevalence of parasites and pathogens in the urban brown rats that we studied, which may suggest that the risk of transmission to people in Helsinki is limited. This could be a reason for the low or non-existent reports of rat-borne pathogen and parasite infections in humans there, but under-diagnosis might also be an explanation, so this could be assessed in the future to further understand the risk posed by urban rats to humans.

## Introduction

In urban areas, pets and domestic animals may acquire certain pathogens from humans and/or transmit them to humans [1]. This can also be the case for wild animals and, although urban environments host limited wildlife, these settings seem to be enriched in species susceptible to human pathogens [2] (but see [3]). Rodents which are often highlighted as a considerable source of human infections [4], have historically, both received zoonotic pathogens from people and transmitted such pathogens to people [5]. In the world, growing urbanisation raises the likelihood for rodents, especially species characterised as pests, to come into contact with people [6]. In addition, as the importance of urban biodiversity gains further recognition, the number of urban green spaces, which can host rodents, may increase, potentially creating more settings for possible transmission events between humans, pets and rodents [7-9].

Brown rats (*Rattus norvegicus*) are one of the most synanthropic mammals in the world and are known carriers of numerous zoonotic pathogens and parasites including helminths (nematodes and cestodes), bacteria and viruses [10]. Thus, they make an interesting species to study in the context of urban pathogen spillovers. Currently, mortality and morbidity caused by rat-borne pathogens and parasites in humans is known to be concentrated in the Global South [11], whereas the same pathogen and parasite species are commonly found in the Global North but with limited public health consequences [10]. While this situation may relate to exposure, risks of zoonotic infections are poorly understood and may evolve due to anthropogenic modifications of the environment and/or climate change [12].

Finland lies on the northernmost continuous distribution area of the brown rat. At such high latitudes, few studies have focused on this species and, in this context, risks of pathogens and parasites occurring in brown rats and of their transmission to humans in Finland are poorly understood. One report from 2005 described two human cases of rat bite fever in the country, which were caused by *Streptobacillus moniliformis* and linked to rats [13]. Finnish rats, however, are likely to carry numerous potentially zoonotic parasites and pathogens.

Within a larger objective of assessing rat-related infectious disease risks in Finland and of further conducting surveys in humans targeting the most relevant underlying pathogen species, the aim of this study was to use stakeholder-collected brown rat samples in the city of Helsinki, Finland [14,15], to investigate whether the rats hosted certain pathogens and parasites, which were potentially zoonotic.

## Methods

### Sample collection

This study was a part of the multidisciplinary Helsinki Urban Rat Project. We acquired rat carcasses between February 2018 and April 2023 from pest management professionals and citizens who collected rats from their kill traps and brought them to our storage freezers along with information on catch date and location. Pest control interventions have previously been suggested for opportunistic sampling of rat-borne pathogens [14], and we used this approach for two main reasons. Firstly, due to the participatory nature of our project, we had extensive collaboration with our stakeholders (e.g. environmental health authorities and property managers), so involving also pest management professionals suited the set-up. Secondly, members of our research project expressed reservations about killing rats for research purposes.

All samples were collected within or next to buildings as lethal pest control is not generally conducted in city parks or other green areas. We mostly limited the samples to the Helsinki City area but also collected additional samples from a waste incineration plant where rats are assumed to arrive not only from Helsinki City but also from a larger area around it (municipalities of Espoo, Hanko, Hyvinkää, Inkoo, Järvenpää, Karkkila, Kauniainen, Kerava, Kirkkonummi, Lohja, Mäntsälä, Nurmijärvi, Pornainen, Raasepori, Sipoo, Siuntio, Tuusula, Vantaa, Vihti and partly Porvoo).

The rat carcasses, which were frozen at − 20 °C, were then defrosted. Upon defrosting, those further included in the study, based on quality inspection, were used to obtain tissue/organs for pathogen or parasite analysis. We set no clear-cut limits for the acceptable time between rat death, and the carcass being deposited in a freezer, as, for example, during the winter-time rat collections, carcasses could be already effectively frozen due to sub-zero ambient temperatures. In general, indoor traps are checked every 24 hours or more frequently to prevent odours. We only included fresh-looking carcasses to limit the effects of decomposition on the analysis. Upon inclusion of the rats in the study, we recorded their sex and body mass.

### Pathogen and parasite investigations

To be targeted by the study, pathogens and parasites had to have been mentioned in the literature as commonly rat-borne and zoonotic [10] and their detection had to be feasible within the project. Their list was as follows: (i) helminths including cestodes and nematodes or other heart-, lung- and gut worms; (ii) bacteria (*Campylobacter jejuni*; other *Campylobacter* spp.; *Leptospira interrogans*); and (iii) viruses (poxviruses, hantaviruses, arenaviruses, rat hepatitis virus (ratHEV)).

We further refer to these as ‘potential’ zoonoses because we do not know whether they cause actual zoonotic infections in Helsinki and whether the pathogens or parasites (or their different strains) are the same as those causing human infections.

For each rat, we collected a piece of colon with a variable amount of faecal matter for *Campylobacter* analysis, a piece of diaphragm and/or thigh muscle for *Trichinella* analysis and tissues from the lung, heart, liver and kidney for virus and *Leptospira *analysis.

#### Helminths

We analysed diaphragm and thigh muscle samples by artificial digestion for *Trichinella* according to European Union Regulation 2015/1375, Annex I, as applicable, accounting for the small size of samples [16]. We inspected the heart, lungs and the whole gastrointestinal tract from the stomach to the large intestine under the microscope for the presence of nematodes, cestodes or other helminths. We morphologically identified any observed helminths to the species level whenever possible. We did not fix or stain tissues. The helminth species were cross-referenced with lists of known brown rat parasites [17] and identified with reference to previous studies [18-20].

#### Bacteria

We homogenised the colon samples using a cotton swab dipped in sterile buffered peptone water and cultured them directly on *Campylobacter*-selective charcoal-cefoperazone–deoxycholate agar (CCDA) plates (Oxoid Ltd., Basingstoke, United Kingdom) that were incubated under microaerobic conditions (5% O_2_, 10% CO_2_, ≤ 10% H_2_, balanced with N_2_; Anoxomat System, Mart Microbiology, the Netherlands) at 41.5 °C for 48–72 hours. One typical colony was confirmed per sample as *Campylobacter* spp. or *C. jejuni* using Gram-stain and genus- and species-specific PCR. We used SsoAdvanced Universal SYBR Green real-time PCR (RT-PCR) Supermix (Bio-Rad, Hercules, California, United States) according to the manufacturer’s instructions with primers 16S-CampyF1 and 16S-CampyR1 [21] or JH0039 and JH0040 [22] (metabion, Planegg, Germany), respectively. We measured fluorescence intensity using the CFX96 Touch RT-PCR Detection System (Bio-Rad) and CFX Maestro Software v.2.3. We considered a sample positive when the quantification cycle was below 30 and a specific melt curve with peak temperature between 78.5 and 79.5 °C was observed.

We detected *Leptospira* by PCR from the kidney samples. We extracted DNA using the Nucleospin Tissue mini kit (Macherey-Nagel, Düren, Germany), followed by quantitative PCR (qPCR) targeting the secY gene of *Leptospira* as previously described [23]. We performed the qPCR using the Agilent Technologies’ AriaMx RT-PCR system and melted the amplified product at 70–94 °C to confirm the identity of the amplified product.

#### Viruses

We tested ratHEV RNA from the liver samples that were homogenised using glass beads and sand in 1 mL TRIzol reagent (Invitrogen, ThermoFisher Scientific, Waltham, Minnesota, United States) with MagNa Lyser (Roche Diagnostics, Rotkreuz, Switzerland). We extracted RNA from the samples with the TRIzol reagent following product instructions and amplified ratHEV RNA in two steps: a hepevirus specific broad-spectrum PCR as a first step, and then a nested PCR protocol, targeting a conserved region of open reading frame (ORF)1 as the second step. For the hepevirus RT-PCR we used Superscript III Platinum One-Step qRT-PCR Kit without carboxyrhodamine (ROX; Invitrogen, Thermo Fisher Scientific) with primers HEV-cs and HEV-cas [24]. Subsequently, the nested PCR used the Platinum Taq DNA polymerase (Invitrogen) with the primers HEV-csn mod and the primer HEV-casn [25]. We performed the PCR reactions with either a ThermoScientific Arktik Thermal Cycler or an MJ Research PTC-200 Peltier Thermal Cycler. For a second approach we confirmed the presence of rat-specific HEV with a higher sensitivity by using a specific RT-qPCR for ratHEV [26] and adapting the protocol to TaqMan Fast Virus 1-Step Master Mix (4X) (Thermo Fisher Scientific). To target the region 5,214–5,286 in the rat/Mu/0685/DEU2010 sequence, we used primers rHEV-F and rHEV-R2 with a rHEV-P2 probe [26] labelled with 6-carboxyfluorescein (6-FAM) at the 5’ end and Black Hole Quencher (BHQ) was used at the 3’ end. We performed RT-PCR using the Agilent Technologies AriaMx RT-PCR system.

We screened samples for antibodies to orthopoxviruses, arenaviruses, and hantaviruses using immunofluorescence assays (IFA), as previously described [27-29]. These assays are developed for specific viruses (Puumala hantavirus, PUUV; Dobrava-Belgrade hantavirus, DOBV; lymphocytic choriomeningitis virus, LCMV; and coxpow virus), but cross-react with other closely related viruses, which is useful when we do not know which particular orthopoxvirus, arenavirus, or hantavirus is present in the samples. As PUUV and DOBV represent different serogroups, we can detect all possible rodent-borne hantaviruses by performing assays on both [27]. The LCMV assay cross-reacts with all Old-World arenaviruses [27,29], and similarly, the cowpox virus assay is highly cross-reactive across orthopoxviruses [28].

Whereas the morphological surveys of helminths, *Campylobacter* culture and *Leptospira* and ratHEV PCR detect acute infections, IFA used for viruses is indicative of past infection.

### Statistical analyses

While the rat carcass sample size was not small, potential statistical analyses were limited by the number of parasite and pathogen species: modelling individual species could easily have led to multiple testing, which in turn reduces the power of analysis. To infer general patterns, we performed one generalised linear mixed model where we used the number of parasite and pathogen species in a sample as a response variable, whereas weight, sex, season and district were used as explanatory variables and year and site nested within district as random variables. We used the lme4 package in R for statistical testing [30]. As lme4 does not report p-values due to the difficulties in estimating degrees of freedom in mixed-effects models, we report Wald t values, where values higher than 1 or lower than −1 indicate substantial differences from zero. We tested the spatial variation in *Campylobacter* presence with the χ^2^ test [31].

## Results

**Figure 1:**
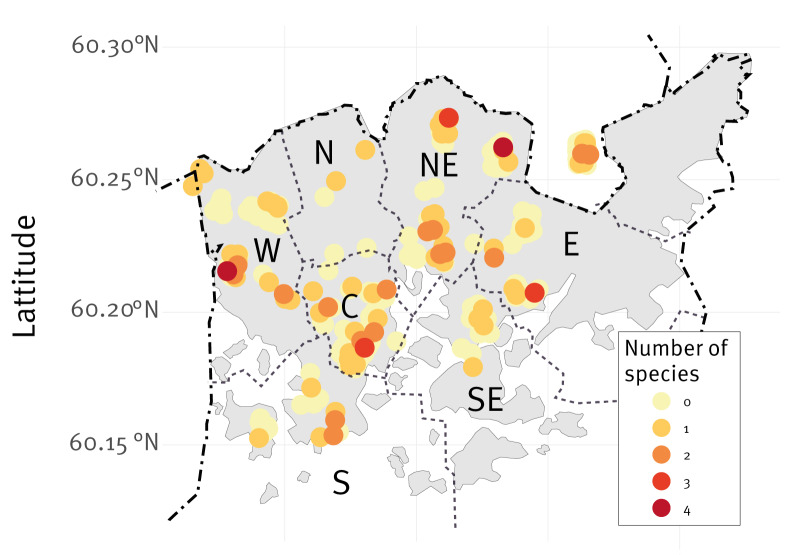
The spatial distribution of samples across the districts of Helsinki, Finland, 2018–2023 (n = 288)

We received a total of 288 rat carcasses that were of adequate quality to conduct at least some analyses (Table 1; Figure 1). Juvenile rats (< 100 g) were more common than adult rats. The median juvenile and adult weights were 54 g and 195 g, respectively, with rat weights ranging from 13.4 g to 368 g. Substantially more samples collected over the study period were from the first half of the year (January–June: n = 231) than from the second half (n = 57), but the sex ratio (146 male/116 female rats) was close to uniform.

**Table 1:** Characteristics of the opportunistic rat sample (*Rattus norvegicus*) within and around Helsinki, Finland, 2018–2023 (n = 288)

Group	Sample size
Weight category	
Juvenile (< 100 g)	170
Adult (> 100 g)	110
Unknown^a^	8
Sex	
Male	146
Female	116
Unknown^a^	26
Collection period of the year	
January–March	122
April–June	109
July–September	20
October–December	37
Year of collection^b^	
2018	20
2019	103
2020	48
2021	44
2022	15
2023	52
Location of collection	
**City of Helsinki**	**245**
Southern major district	17
Western major district	53
Central major district	72
Northern major district	4
North-eastern major district	31
South-eastern major district	22
Eastern major district	46
**Incineration plant**	**43**
Total	288

^a^ Some individuals could not reliably be sexed due to small size, whereas some could not be reliably weighed, as they were missing a substantial (but for the purposes of this study non-considerable) part of the body.

^b^ For six rats, the year of collection was not recorded.

C: Central major district; E: Eastern major district; N: Northern major district; NE: North-eastern major district; S: Southern major district; SE: South-eastern major district; W: Western major district.

For overall pathogen and parasite diversity in the samples, red refers to the highest species richness in individual samples, whereas a lighter colour indicates lower species richness. The locations were generalised to mask the accurate locations of the sampling sites. Gray represents the land area within city limits. Samples from the incineration plant are those that fall outside of city borders, which are represented by a thick dotted line.

We observed most of the pathogens and parasites under survey, except for *Trichinella* and *Angiostrongylus* or other lung- and heart worms (Table 2). As the sample size from the waste incineration plant (i.e. ‘outside’ of the city of Helsinki) was smaller, the prevalences of the two locations cannot be reliably compared. The only *H. diminuta* infected individuals were found from a rat at the waste incineration plant.

**Table 2:** Pathogen and parasite prevalences in urban brown rats (*Rattus norvegicus*) within and around Helsinki, Finland, 2018–2023 (n = 288)

Pathogens or parasites		All rats			Rats within city of Helsinki								
					Juvenile			Adults			Both		
		Numbers		% (95%CI)	Numbers		% (95%CI)	Numbers		% (95%CI)	Numbers		% (95%CI)
		+	Successfully tested		+	Successfully tested		+	Successfully tested		+	Successfully tested	
Viruses	Poxviruses	11	211	5.2 (2.6–8.7)	1	111	0.9 (0–3.6)	10	84	11.9 (5.7–20.5)	11	195	5.6 (2.8–9.5)
	Hantaviruses	11	211	5.2 (2.6–8.7)	2	111	1.8 (0.2–5.2)	9	84	10.7 (4.9–18.9)	11	195	5.6 (2.8–9.5
	Arenaviruses	6	211	2.8 (1.0–5.6)	1	111	0.9 (0–3.6)	5	84	6.0 (1.9–12.49)	6	195	3.1 (1.1–6.0)
	Rat hepatitis E virus	4	216	1.8 (0.5–4.1)	1	114	0.9 (0.0–3.5)	2	96	2.1 (0.2–5.6)	3	200	1.5 (0.3–3.7)
Bacteria	*Campylobacter jejuni*	17	259	6.6 (3.8–10.1)	2	136	1.5 (0.1–4.2)	13	84	15.4 (8.2–25.1)	14	220	6.4 (3.5–10.1)
	Other *Campylobacter* spp.	2	259	0.8 (0.1–2.2)	2	136	1.5 (0.1–4.2))	0	84	0 (0–1.5)	2	220	0.9 (0–2.6)
	*Leptospira interrogans*	2	163	1.2 (0.1–3.5)	0	82	0 (0–1.5)	2	65	3.1 (0.3–8.9)	2	147	1.4 (0.1–3.9)
Cestodes	*Hymenolepis nana*	28	288	9.7 (6.3–13.3)	11	151	7.3 (3.6–12.2)	13	94	13.7 (7.3–22.4)	24	245	9.8 (6.3–14.1)
	*Hymenolepis diminuta*	1	288	0.3 (0.0–1.3)	0	151	0 (0–0–8)	0	94	0 (0–1.3)	0	245	0 (0–0.5)
Nematodes	*Heterakis spumosa*	15	288	5.1 (2.8–8.0)	4	151	2.6 (0.7–5.9)	11	94	11.7 (5.8–19.7)	15	245	6.1 (3.4–9.6)
	*Syphacia* sp.	11	288	3.7 (1.9–6.3)	5	151	3.3 (1.1–6.9)	5	94	5.3 (1.7–11.0)	10	245	4.1 (1.9–7.0)
	*Nippostrongylus* sp.	5	288	1.7 (0.6–4.2)	0	151	0 (0–0.8)	5	94	5.3 (1.7–11.0)	5	245	2.0 (0.6–4.2)
	*Trichinella* spp.	0	257	0 (0–0.5)	0	138	0 (0–0.9)	0	84	0 (0–1.5)	0	222	0 (0–0.6)
	*Angiostrongylus* spp. or other heart and lung worms	0	288	0 (0–0.4)	0	151	0 (0–0.8)	0	94	0 (0–1.3)	0	245	0 (0–0.5)

CI: confidence intervals; max.: maximum; +: positive.

**Figure 2:**
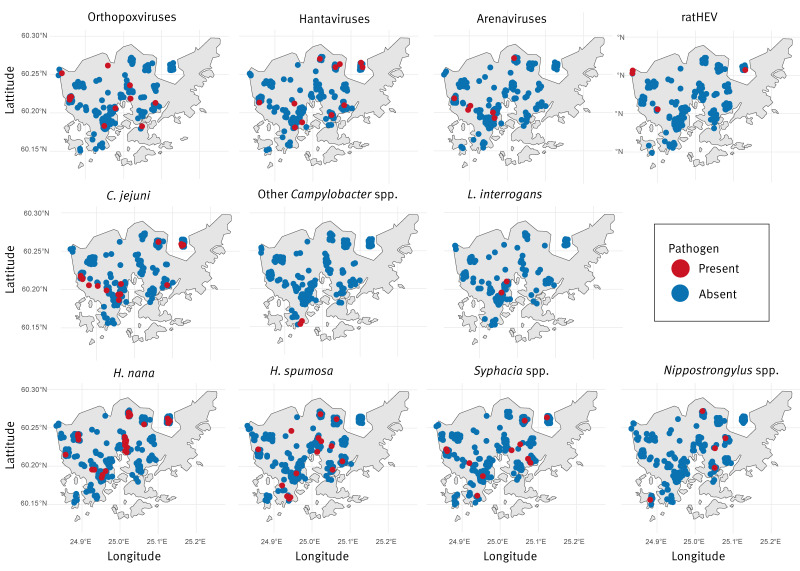
The occurrence in urban brown rats (*Rattus norvegicus*) of each pathogen or parasite plotted on a map of Helsinki, Finland, 2018–2023

Prevalences are given in percentages with 95% CIs. Data from the waste incineration plant are not shown, as the sample size is low (max. n = 35). The spatial distribution of individual pathogens is shown in Figure 2.

*C. jejuni*: *Campylobacter jejuni*; *H. nana*: *Hymenolepis nana*; *H. spumosa*: *Heterakis spumosa*; *L. interrogans*: *Leptospira interrogans;* ratHEV: rat hepatitis E virus.

For individual pathogens and parasites red shows presence and blue shows absence. The city area is shown in grey. On the axes, N and E respectively indicate the latitude (north) and longitude (east). The locations were generalised to mask the accurate locations of the sampling sites. Samples from the waste incineration plant are those that fall outside of city borders.

When considering both acute infections (i.e. helminths, *Campylobacter*, *Leptospira* and ratHEV) and past infections detected by IFA (orthopoxviruses, arenaviruses, and hantaviruses), 7.1% (21/295) of all rat individuals had or had had multiple infections (Figure 1), with extreme cases where two individuals had four different parasites and pathogens each: one had detected *H. nana*, *H. spumosa*, cultured *C. jejuni* and seropositivity for hantavirus, and the other had detected *H. nana*, cultured *C. jejuni* and seropositivity for arenavirus and hantavirus.

The heavier rats were more likely to have parasites and pathogens, whereas neither sex nor season had a significant effect on richness of parasites and pathogens (Table 3). Rats from the Northern and South-eastern districts were less likely to have parasites and pathogens than those from other districts, whereas rats from the North-eastern and Southern districts were more likely to be infected (Table 3; Figure 1).

**Table 3:** Estimates from the mixed effects modelling for parasite and pathogen richness in urban brown rats (*Rattus norvegicus*) sampled within and around Helsinki, Finland, 2018–2023 (n = 288)

Variable	Categories	Estimate	Standard Error	Wald t value^a^
Intercept	NA	− 0.118	0.238	− 0.50
Weight	NA	0.003	0.001	6.07
Sex	Female	0.087	0.220	0.39
	Male	0.137	0.213	0.64
Season	Spring	0.052	0.140	0.37
	Summer	0.064	0.192	0.33
	Winter	0.054	0.135	− 0.40
Location	Eastern district	0.085	0.136	0.62
	Northern district	− 0.489	0.476	− 1.03
	North-eastern district	0.247	0.151	1.63
	Southern district	0.217	0.189	1.15
	South-eastern district	− 0.271	0.174	− 1.56
	Western district	0.117	0.122	0.97
	Incineration plant	− 0.049	0.137	− 0.36

NA: not applicable.

^a^ Wald t values over 1 or below − 1 indicate substantial differences from the baseline. The baseline case is a rat of unknown sex sampled in autumn in the Central district.

## Discussion

In this study we found low parasite and pathogen prevalences among brown rats in Helsinki compared with other studies in Europe. For example, in different European cities, prevalence in brown and/or black rat population of *H. diminuta* varied from 1.2 to 36.3% [32-37] according to estimations between 1975 and 2017, while based on work between 2010 and 2017, the prevalence of *H. spumosa* ranged from 35 to 82.5% [33-38], and that of *Nippostrongylus brasiliensis* from 6.2 to 46.0% [33-37]. In a publication from 2010, ratHEV was detected through RT-PCR in 0 to 27.2% of rats when sampling across continental Europe [39], whereas Seoul hantavirus (SEOV) prevalence reported in 2009 in wild rats across Flanders, Belgium was 15 to 33% [40]. *Leptospira* seroprevalences have been found to vary in Europe between 1.2% and 100% in urban or peri-urban environments in studies conducted between 1995 and 2016 [41-54]. Thus, rats in Helsinki seem to be in the lower bounds of the prevalence ranges for viral and bacterial pathogens and helminths.

The temporal trend in rat pathogens also appears to be declining: two of the taxa that we surveyed had been investigated previously in the Helsinki region: *Leptospira* prevalence was 43.5% in 1952–53 [55], whereas the prevalence was only 1.2% in our sample. The comparison is not straightforward, as the sampling method is not described in the previous study and *Leptospira* infections were earlier detected serologically, but the difference seems stark. Also, human *Leptospira* cases have been rare in Finland, overall, with 16 reported allochthonous cases between 2011 and 2023, of whom the last one in 2016 [56]. *Trichinella spiralis* prevalence in rats at the Helsinki Zoo was 12% in 1965 [57], and the overall *Trichinella* prevalence in dump pits in the Helsinki area was 19% in 1994–2000 [58], whereas we found no occurrences in our sample. The general decrease of sylvatic *T. spiralis* in Finland is attributed to the absence of spillover from the domestic cycle [59]. Other *Trichinella* species prevalent in Finland do not readily infect rats [60]. Rats are currently usually not considered an important reservoir for *T. spiralis*, but rather an indicator of its presence in the environment [61].

*Campylobacter jejuni* prevalence in adult rats in the Helsinki City area (15%) was comparable to levels previously reported around animal-production farms in Finland (20%, n = 10 [62]), lower than on pig farms in France (40%, n = 40 [63]), yet higher than on pig and chicken farms in Sweden (3%, n = 58 [64]). In urban rats in New York city and in fish markets and restaurants in Tokyo, lower prevalences of *C. jejuni* and *C. coli* (4%, 5% and 0%, respectively) have been reported [65,66] whereas rats in the Lyon sewage system had a prevalence of 18% (n = 92 rats caught in 1982) [67]. Interestingly, the older parts of the Helsinki City have a mixed sewage system, i.e. both household waste and rainwater run in the same sewage system. This mixed system is thought to be beneficial for rats as they can easily access it from rainwater drains and then forage among household waste. This also leads to the questions whether transmission is occurring between rats and humans or rather vice versa, as this setting also potentially allows for anthroponotic infections in rats if and when the latter come into contact with human faeces. It is difficult to track how many, and which individual, rats move in the sewage system and conclusively deduce whether *Campylobacter* infections were more common in these areas (10 positives out of 128 in mixed sewer area vs 4 of 130 in other areas; χ^2^_1_ = 2.81, p = 0.09). Nevertheless, this calls for more detailed studies on the infection risks that humans pose to rats, and to understand the importance of this transmission route regarding zoonosis persistence and spread in the urban environment.

No previous surveys have been conducted on seroprevalence in rats in Helsinki and our results suggest the first evidence for the occurrence of rat-associated Seoul hantavirus in Finland. This detection needs to be followed up with genetic characterisation. On a positive note, we found no signs of *Angiostrongylus* or other lung- or heart-related nematodes. The first autochthonous cases of rat heart worm *Angiostrongylus cantonensis* were recorded in *R. rattus* and *R. norvegicus* in 2021 in Valencia, Spain [68], thus calling for continued surveillance of this zoonotic parasite. *A. cantonensis* is currently spreading across the world and it has been limited to subtropical and tropical regions [69]. As the biotic and abiotic limitations of its spread are to date unknown [70], it remains an open question whether the parasite could survive in Helsinki rats.

Numerous factors influence the transmission and spread of rat-borne parasites and pathogens, all of which could explain why the prevalences in our survey appear comparably low. Indeed, the spatial heterogeneity of within-rat communities is known to be pervasive across various scales [71]. There are reasons to expect that no single explanation would cover all the different species as the studied pathogens and parasites are transmitted from individual to individual (viral pathogens, *Leptospira*, *Campylobacter, Trichinella*), via the environment (*Leptospira*, *Campylobacter*, *Nippostrongylus*, *Heterakis*, *Syphacia*) or through intermediate hosts (*Hymenolepis*, *Trichinella*). They have varying levels of host specificity and competence, with some mainly infecting brown rats, while others circulate in a wider range of local rodents and other mammals. Different transmission modes, reservoir host communities and survivability in the environment lead to differing drivers for these zoonoses.

Observed communities always result from biogeographic events where rat population connectivity and individuals’ movements shape pathogen spread, and limited rat movement has oftentimes been suggested as a reason for variation in pathogen communities across the scales [72]. Spatial discontinuities and bottlenecks in rat populations can also be caused, for example, by pest management operations [73] or adverse environmental events, such as cold winters or dry summers [74]. Indeed, Helsinki is a northern city and has comparably cold winters in comparison to many European cities, which can cause rat population bottlenecks. In contrast, Helsinki has no coordinated rat control plans, and private companies work on a site-by-site basis [75]. Interestingly, the effect of pest management is poorly understood. While lethal rat control is performed to reduce pathogen and parasite circulation, there is evidence only to suggest the contrary [76]. Anecdotally, the sites where the most rat individuals were collected in this study also had higher parasite and pathogen richness, but this needs to be studied more carefully. An assessment of rat population size, its seasonal variation and the effect of pest management operations is under way in the Helsinki Urban Rat Project.

The future trends of rat pathogen and parasite prevalences are difficult to assess. Larger-scale green areas have diminished in Helsinki [77], whereas the effect of small-scale greenery, such as the inner courtyards of buildings, on rat populations is poorly known. While current population densities are unknown, high rat population densities have historically been linked, for example, to waste dumps and landfills which have all been closed in the city of Helsinki [78]. Climate change could increase mean temperature and rainfall, both of which could likely affect rat population sizes and the transmission of parasites and pathogens.

The reliability regarding the observation that the prevalence of rat-borne pathogens is low is supported by the lack of diagnosed rat-associated human cases of these zoonoses. Indeed, we have found only two described cases in the literature of rat-borne infections in humans in Finland in recent decades, both outside of Helsinki [13]. While the lack of known cases could be due to actual low numbers of zoonotic infections, our results show that potentially zoonotic pathogens and parasites are present in urban rats in Helsinki. Thus, there is a possibility of undiagnosed rat-borne infections. For example, Seoul hantavirus infections can present in a similar manner as Puumala hantavirus infections for which the annual incidence is ca 31 cases/100,000 person years (mostly based on clinical symptoms) [79]. To assess the actual risks caused by these rat-borne pathogens and parasites, better detection of human cases is needed.

Biased sampling of rats across the city is another possible explanation for the untypical prevalences. Due to the expectation of highly heterogenous rat-associated pathogen and parasite communities, representative sampling is difficult on a city-wide scale and as rats are difficult to catch at any scale [80]. As pest management companies are contacted commonly only when rats are encountered on urban sites, we would expect that our samples are from sites with higher rat population densities than in the city overall. Thus, the rats included in this study are likely to overrepresent a situation with numerous rats or larger rat colonies in contrast to sites that have fewer rats. The samples were biased towards the winter months (November to April), as rat carcasses were better preserved in traps at this time. Our preliminary data suggest that also rat population densities might represent at their annual lowest during the winter. Nevertheless, a previous study has shown that carcass collection by pest management company broadly corresponds to random sampling [15].

Due to different methods, we expect that the detection analysis of different pathogens and parasites have, for example, varying reliabilities and rates of false negatives. For instance, helminths are very reliably detected as only carcasses with no intestinal decomposition were used. In contrast, the quantity of faecal matter varied in the intestinal samples, and this could not be standardised, likely affecting *Campylobacter* spp. detection. Also freezing the carcasses before analysis potentially reduced the number of live *Campylobacter* cells and thus culture-positive sample numbers. For viruses, we mostly used antibody identifications which are quite reliable even in older carcasses. We would expect sample quality to be more compromised when it comes to PCR or genome sequencing methods, especially in relation to RNA viruses, such as ratHEV.

Even though Helsinki may have lower overall prevalence of certain rat-borne pathogens and parasites than other previously surveyed cities in Europe, it is important not to interpret our results as an indication of the local rat-borne zoonotic risk. As mentioned, rat-borne pathogen communities vary substantially, and even in situations of true low overall prevalence across the city, local prevalence at individual sites can be very high. Similarly, the probability of rat-borne microbes being transmitted to humans also depends on several other risk factors other than rat population-level prevalences, such as exposure [81]. It should also be noted that we do not know whether these pathogens and parasites could cause infections in humans and thus assessing the risk to humans is difficult. Further work is under way to especially identify viral species and genotype *Campylobacter* spp. with whole genome sequencing. 

## Conclusion

Here, we present a survey of potentially zoonotic parasites and pathogens in urban rats in Helsinki, Finland, during 2018 to 2023. While several pathogens and parasites encountered in other cities in Europe were found, we also noted an apparent absence of rat lung and heartworms and *Trichinella* nematodes. In general, parasite and pathogen prevalences appeared low compared with other European cities. Our survey suggests that low or non-existent diagnosis levels of rat-borne pathogen and parasite infections in humans may partly be due to a limited transmission in rats.

## References

[1] Hassell JM Muloi DM VanderWaal KL Ward MJ Bettridge J Gitahi N Epidemiological connectivity between humans and animals across an urban landscape. Proc Natl Acad Sci USA. 2023;120(29):e2218860120. 10.1073/pnas.2218860120 37450494 PMC10629570

[2] Gibb R Redding DW Chin KQ Donnelly CA Blackburn TM Newbold T Zoonotic host diversity increases in human-dominated ecosystems. Nature. 2020; 584(7821):398-402. 10.1038/s41586-020-2562-8 32759999

[3] Albery GF Carlson CJ Cohen LE Eskew EA Gibb R Ryan SJ Urban-adapted mammal species have more known pathogens. Nat Ecol Evol. 2022;6(6):794-801. 10.1038/s41559-022-01723-0 35501480

[4] Meerburg BG Singleton GR Kijlstra A . Rodent-borne diseases and their risks for public health. Crit Rev Microbiol. 2009;35(3):221-70. 10.1080/10408410902989837 19548807

[5] Himsworth CG Parsons KL Jardine C Patrick DM . Rats, cities, people, and pathogens: a systematic review and narrative synthesis of literature regarding the ecology of rat-associated zoonoses in urban centers. Vector Borne Zoonotic Dis. 2013;13(6):349-59. 10.1089/vbz.2012.1195 23590323

[6] Rothenburger JL Himsworth CH Nemeth NM Pearl DL Jardine CM . Environmental Factors and Zoonotic Pathogen Ecology in Urban Exploiter Species. EcoHealth. 2017;14(3):630-41. 10.1007/s10393-017-1258-5 28631116

[7] Shackleton CM Ruwanza S Sinasson Sanni GK Bennett S De Lacy P Modipa R Unpacking Pandora’s Box: Understanding and Categorising Ecosystem Disservices for Environmental Management and Human Wellbeing. Ecosystems (N Y). 2016;19(4):587-600. 10.1007/s10021-015-9952-z

[8] Vanwambeke SO Linard C Gilbert M . Emerging challenges of infectious diseases as a feature of land systems. Curr Opin Environ Sustain. 2019;38:31-36. 10.1016/j.cosust.2019.05.005

[9] de Cock MP de Vries A Fonville M Esser HJ Mehl C Ulrich RG Increased rat-borne zoonotic disease hazard in greener urban areas. Sci Total Environ. 2023;896:165069. 10.1016/j.scitotenv.2023.165069 37392874

[10] Strand TM Lundkvist Å . Rat-borne diseases at the horizon. A systematic review on infectious agents carried by rats in Europe 1995-2016. Infect Ecol Epidemiol. 2019;9(1):1553461. 10.1080/20008686.2018.1553461 30834071 PMC6394330

[11] Global Burden of Disease Collaborative Network. Global Burden of Disease Study 2019 (GBD 2019). Seattle, United States: Institute for Health Metrics and Evaluation; 2020.

[12] Estrada-Peña A Ostfeld RS Peterson AT Poulin R de la Fuente J . Effects of environmental change on zoonotic disease risk: an ecological primer. Trends Parasitol. 2014;30(4):205-14. 10.1016/j.pt.2014.02.003 24636356

[13] Syrjänen J Mustonen J Vapalahti O Henttonen H Vaheri A . Jyrsijöiden levittämät sairaudet Suomessa. [Rodent dissemination of diseases in Finland]. Duodecim. 2005;121(3):295-302. 15787287

[14] Strand TM Pineda S Backhans A Jakobsen F Råsbäck T Lõhmus M Detection of *Leptospira* in Urban Swedish Rats: Pest Control Interventions as a Promising Source of Rats Used for Surveillance. Vector Borne Zoonotic Dis. 2019;19(6):414-20. 10.1089/vbz.2017.2262 30785372

[15] Robinson SJ Finer R Himsworth CG Pearl DL Rousseau J Weese JS Evaluating the utility of pest control sourced rats for zoonotic pathogen surveillance. Zoonoses Public Health. 2022;69(5):468-74. 10.1111/zph.12936 35253370

[16] European Commission. Commission implementing regulation (EU) 2015/1375 of 10 August 2015 laying down specific rules on official controls for Trichinella in meat. 2015. Available from: http://www.iss.it/crlp/index.php

[17] Gliga DS Pisanu B Walzer C Desvars-Larrive A . Helminths of urban rats in developed countries: a systematic review to identify research gaps. Parasitol Res. 2020;119(8):2383-97. 10.1007/s00436-020-06776-3 32607706 PMC7366588

[18] Ribas A de Bellocq JG Ros A Ndiaye PI Miquel J . Morphometrical and genetic comparison of two nematode species: H. spumosa and H. dahomensis (Nematoda, Heterakidae). Acta Parasitol. 2013;58(3):389-98. 10.2478/s11686-013-0156-4 23990438

[19] Durette-Desset MC . Le genre Nippostrongylus Lane, 1923, (Nématode-Héligmosomatidé). [The genus Nippostrongylus Lane, 1923 (Nematoda-Heligmosomatidae)]. Ann Parasitol Hum Comp. 1970;45(6):815-21. 10.1051/parasite/1970456815 5535153

[20] Khalil LF, Jones A, Bray RA. Keys to the cestode parasites of vertebrates. Wallingford: CAB International; 1994.

[21] de Boer P Rahaoui H Leer RJ Montijn RC van der Vossen JMBM . Real-time PCR detection of Campylobacter spp.: A comparison to classic culturing and enrichment. Food Microbiol. 2015;51:96-100. 10.1016/j.fm.2015.05.006 26187833

[22] Chaban B Musil KM Himsworth CG Hill JE . Development of cpn60-based real-time quantitative PCR assays for the detection of 14 Campylobacter species and application to screening of canine fecal samples. Appl Environ Microbiol. 2009;75(10):3055-61. 10.1128/AEM.00101-09 19304828 PMC2681665

[23] Ahmed A Engelberts MFM Boer KR Ahmed N Hartskeerl RA . Development and validation of a real-time PCR for detection of pathogenic leptospira species in clinical materials. PLoS One. 2009;4(9):e7093. 10.1371/journal.pone.0007093 19763264 PMC2740861

[24] Johne R Plenge-Bönig A Hess M Ulrich RG Reetz J Schielke A . Detection of a novel hepatitis E-like virus in faeces of wild rats using a nested broad-spectrum RT-PCR. J Gen Virol. 2010;91(Pt 3):750-8. 10.1099/vir.0.016584-0 19889929

[25] Widén F Ayral F Artois M Olofson AS Lin J . PCR detection and analysis of potentially zoonotic Hepatitis E virus in French rats. Virol J. 2014;11(1):90. 10.1186/1743-422X-11-90 24886183 PMC4040512

[26] Johne R Dremsek P Kindler E Schielke A Plenge-Bönig A Gregersen H Rat hepatitis E virus: geographical clustering within Germany and serological detection in wild Norway rats (Rattus norvegicus). Infect Genet Evol. 2012;12(5):947-56. 10.1016/j.meegid.2012.02.021 22554648

[27] Kallio-Kokko H Laakkonen J Rizzoli A Tagliapietra V Cattadori I Perkins SE Hantavirus and arenavirus antibody prevalence in rodents and humans in Trentino, Northern Italy. Epidemiol Infect. 2006;134(4):830-6. 10.1017/S0950268805005431 16371172 PMC2870443

[28] Kinnunen PM Henttonen H Hoffmann B Kallio ER Korthase C Laakkonen J Orthopox virus infections in Eurasian wild rodents. Vector Borne Zoonotic Dis. 2011;11(8):1133-40. 10.1089/vbz.2010.0170 21453121

[29] Forbes KM Voutilainen L Jääskeläinen A Sironen T Kinnunen PM Stuart P Serological survey of rodent-borne viruses in finnish field voles. Vector Borne Zoonotic Dis. 2014;14(4):278-83. 10.1089/vbz.2013.1526 24689532 PMC3993079

[30] Bates D Mächler M Bolker B Walker S . Fitting linear mixed-effects models using lme4. J Stat Softw. 2015;67(1). 10.18637/jss.v067.i01

[31] Pearson KX . On the criterion that a given system of deviations from the probable in the case of a correlated system of variables is such that it can be reasonably supposed to have arisen from random sampling. Lond Edinb Dublin Philos Mag J Sci. 1900;50(302):157-75. 10.1080/14786440009463897

[32] Dyk V Tilc K Hanuskova Z . Endoparasites of the rat (Rattus norvegicus, Berkenhout, 1769) in old and modern city districts and in the zoological garden. Acta Vet Brno. 1975;44:245-51.

[33] Galán-Puchades MT Sanxis-Furió J Pascual J Bueno-Marí R Franco S Peracho V First survey on zoonotic helminthosis in urban brown rats (Rattus norvegicus) in Spain and associated public health considerations. Vet Parasitol. 2018;259(June):49-52. 10.1016/j.vetpar.2018.06.023 30056983

[34] Desvars-Larrive A Hammed A Hodroge A Berny P Benoît E Lattard V Population genetics and genotyping as tools for planning rat management programmes. J Pest Sci. 2019;92(2):691-705. 10.1007/s10340-018-1043-4

[35] Milazzo C Ribas A Casanova JC Cagnin M Geraci F Di Bella C . Helminths of the brown rat (Rattus norvegicus) (Berkenhout, 1769) in the city of Palermo, Italy. Helminthologia. 2010;47(4):238-40. 10.2478/s11687-010-0037-4

[36] Kataranovski M Mirkov I Belij S Popov A Petrović Z Gačić Z Intestinal helminths infection of rats (Ratus norvegicus) in the Belgrade area (Serbia): the effect of sex, age and habitat. Parasite. 2011;18(2):189-96. 10.1051/parasite/2011182189 21678796 PMC3671415

[37] Franssen F Swart A van Knapen F van der Giessen J . Helminth parasites in black rats (Rattus rattus) and brown rats (Rattus norvegicus) from different environments in the Netherlands. Infect Ecol Epidemiol. 2016;6(1):31413. 10.3402/iee.v6.31413 27193418 PMC4871897

[38] McGarry JW Higgins A White NG Pounder KC Hetzel U . Zoonotic helminths of urban brown rats (Rattus norvegicus) in the UK: neglected public health considerations? Zoonoses Public Health. 2015;62(1):44-52. 10.1111/zph.12116 24661776

[39] Ryll R Bernstein S Heuser E Schlegel M Dremsek P Zumpe M Detection of rat hepatitis E virus in wild Norway rats (Rattus norvegicus) and Black rats (Rattus rattus) from 11 European countries. Vet Microbiol. 2017;208:58-68. 10.1016/j.vetmic.2017.07.001 28888650

[40] Heyman P Baert K Plyusnina A Cochez C Lundkvist A Esbroeck MV Serological and genetic evidence for the presence of Seoul hantavirus in Rattus norvegicus in Flanders, Belgium. Scand J Infect Dis. 2009;41(1):51-6. 10.1080/00365540802459994 18821445

[41] Boey K Shiokawa K Rajeev S . Leptospira infection in rats: A literature review of global prevalence and distribution. PLoS Negl Trop Dis. 2019;13(8):e0007499. 10.1371/journal.pntd.0007499 31398190 PMC6688788

[42] Zilber AL Belli P Artois M Kodjo A Djelouadji Z . First Observation of *Leptospira interrogans* in the Lungs of *Rattus norvegicus.* BioMed Res Int. 2016;2016:9656274. 10.1155/2016/9656274 27800495 PMC5069378

[43] Ayral F Zilber AL Bicout DJ Kodjo A Artois M Djelouadji Z . Distribution of leptospira interrogans by multispacer sequence typing in urban Norway rats (Rattus norvegicus): A survey in France in 2011-2013. PLoS One. 2015;10(10):e0139604. 10.1371/journal.pone.0139604 26447693 PMC4598087

[44] Ayral F Artois J Zilber AL Widén F Pounder KC Aubert D The relationship between socioeconomic indices and potentially zoonotic pathogens carried by wild Norway rats: a survey in Rhône, France (2010-2012). Epidemiol Infect. 2015;143(3):586-99. 10.1017/S0950268814001137 24838220 PMC4411646

[45] Jensen PM Magnussen E . Is it too cold for Leptospira interrrogans transmission on the Faroese Islands? Infect Dis (Lond). 2016;48(2):156-60. 10.3109/23744235.2015.1092579 26442766

[46] Ferreira AS Costa P Rocha T Amaro A Vieira ML Ahmed A Direct detection and differentiation of pathogenic Leptospira species using a multi-gene targeted real time PCR approach. PLoS One. 2014;9(11):e112312. 10.1371/journal.pone.0112312 25398140 PMC4232388

[47] Heuser E Fischer S Ryll R Mayer-Scholl A Hoffmann D Spahr C Survey for zoonotic pathogens in Norway rat populations from Europe. Pest Manag Sci. 2017;73(2):341-8. 10.1002/ps.4339 27299665

[48] Collares-Pereira M Mathias ML Santos-Reis M Ramalhinho MG Duarte-Rodrigues P . Rodents and Leptospira transmission risk in Terceira island (Azores). Eur J Epidemiol. 2000;16(12):1151-7. 10.1023/A:1010916132497 11484805

[49] Vitale M Agnello S Chetta M Amato B Vitale G Bella CD Human leptospirosis cases in Palermo Italy. The role of rodents and climate. J Infect Public Health. 2018;11(2):209-14. 10.1016/j.jiph.2017.07.024 28802826

[50] Broom JC Gibson EA . Infection rates of Rattus norvegicus with Leptospira icterohaemorrhagiae in Great Britain. I. A rural area in Carmarthenshire, Wales. J Hyg (Lond). 1953;51(3):416-25. 10.1017/S0022172400015837 13096748 PMC2217730

[51] Krøjgaard LH Villumsen S Markussen MDK Jensen JS Leirs H Heiberg AC . High prevalence of Leptospira spp. in sewer rats (Rattus norvegicus). Epidemiol Infect. 2009;137(11):1586-92. 10.1017/S0950268809002647 19393116

[52] Collares-Pereira M Korver H Terpstra WJ Santos-Reis M Ramalhinho MG Mathias ML First epidemiological data on pathogenic leptospires isolated on the Azorean islands. Eur J Epidemiol. 1997;13(4):435-41. 10.1023/A:1007383405833 9258550

[53] Aviat F Blanchard B Michel V Blanchet B Branger C Hars J Leptospira exposure in the human environment in France: A survey in feral rodents and in fresh water. Comp Immunol Microbiol Infect Dis. 2009;32(6):463-76. 10.1016/j.cimid.2008.05.004 18639932

[54] Millán J Cevidanes A Chirife AD Candela MG León-Vizcaíno L . Risk factors of Leptospira infection in Mediterranean periurban micromammals. Zoonoses Public Health. 2018;65(1):e79-85. 10.1111/zph.12411 29058382

[55] Rislakki V Salminen A . Investigations of leptospirosis in rats in Finland. Acta Pathol Microbiol Scand. 1955;37(1):121-31. 10.1111/j.1699-0463.1955.tb00927.x 14398273

[56] Raulo S, Kyyrö J, Gadd T, Hallanvuo S, Hietanen P, Oksanen A, et al. Suomen zoonoositilanne ja riskit yhteisen terveyden näkökulmasta: Yhteenveto zoonoosien suuntauksista ja lähteistä 2011-2021. [Finland's zoonotic situation and risks from the perspective of One Health: Summary of trends and sources of zoonoses 2011-2021]. Helsinki; 2023.

[57] Tiainen OA . Occurrence of Trichinella spiralis Ow. (Nematoda, Trichinelloidea) in rats at the Helsinki City Zoological Gardens. Ann Zool Fenn. 1966;3:4.

[58] Mikkonen T Valkama J Wihlman H Sukura A . Spatial variation of Trichinella prevalence in rats in Finnish waste disposal sites. J Parasitol. 2005;91(1):210-3. 10.1645/GE-3230RN 15856908

[59] Oksanen A Interisano M Isomursu M Heikkinen P Tonanzi D Oivanen L Trichinella spiralis prevalence among wildlife of a boreal region rapidly reduced in the absence of spillover from the domestic cycle. Vet Parasitol. 2018;262:1-5. 10.1016/j.vetpar.2018.09.002 30389004

[60] Malakauskas A Kapel CMO Webster P . Infectivity, persistence and serological response of nine Trichinella genotypes in rats. Parasite. 2001;8(2) Suppl;S216-22. 10.1051/parasite/200108s2216 11484361

[61] Pozio E . Factors affecting the flow among domestic, synanthropic and sylvatic cycles of Trichinella. Vet Parasitol. 2000;93(3-4):241-62. 10.1016/S0304-4017(00)00344-7 11099840

[62] Olkkola S Rossi M Jaakkonen A Simola M Tikkanen J Hakkinen M Host-Dependent Clustering of *Campylobacter* Strains From Small Mammals in Finland. Front Microbiol. 2021;11:621490. 10.3389/fmicb.2020.621490 33584588 PMC7873845

[63] Le Moine V Vannier P Jestin A . Microbiological Studies of Wild Rodents in Farms as Carriers of Pig Infectious Agents. Prev Vet Med. 1987;4(5-6):399-408. 10.1016/0167-5877(87)90026-2

[64] Backhans A Jacobson M Hansson I Lebbad M Lambertz ST Gammelgård E Occurrence of pathogens in wild rodents caught on Swedish pig and chicken farms. Epidemiol Infect. 2013;141(9):1885-91. 10.1017/S0950268812002609 23174339 PMC9151424

[65] Firth C Bhat M Firth MA Williams SH Frye MJ Simmonds P Detection of zoonotic pathogens and characterization of novel viruses carried by commensal Rattus norvegicus in New York City. MBio. 2014;5(5):e01933-14. 10.1128/mBio.01933-14 25316698 PMC4205793

[66] Kato Y Nakai Y Matsushita M Takagi Y Kohzaki KI Kaneuchi C . Detection of Salmonella and Campylobacter from rats trapped at restaurants and a fish market. Nippon Juishikai Zasshi. 1999;52(3):194-7. 10.12935/jvma1951.52.194

[67] Seguin B Boucaud-Maître Y Quenin P Lorgue G . Bilan épidémiologique d’un échantillon de 91 rats (Rattus norvegicus) capturés dans les égouts de Lyon. [Epidemiologic evaluation of a sample of 91 rats (Rattus norvegicus) captured in the sewers of Lyon]. Zentralbl Bakteriol Mikrobiol Hyg A. 1986;261(4):539-46. 10.1016/S0176-6724(86)80088-8 3532637

[68] Galán-Puchades MT Gómez-Samblás M Osuna A Sáez-Durán S Bueno-Marí R Fuentes MV . Autochthonous Angiostrongylus cantonensis lungworms in urban rats, Valencia, Spain, 2021. Emerg Infect Dis. 2022;28(12):2564-7. 10.3201/eid2812.220418 36418005 PMC9707565

[69] Barratt J Chan D Sandaradura I Malik R Spielman D Lee R Angiostrongylus cantonensis: a review of its distribution, molecular biology and clinical significance as a human pathogen. Parasitology. 2016;143(9):1087-118. 10.1017/S0031182016000652 27225800

[70] Morgan ER Modry D Paredes-Esquivel C Foronda P Traversa D . Angiostrongylosis in Animals and Humans in Europe. Pathogens. 2021;10(10):1236. 10.3390/pathogens10101236 34684185 PMC8538298

[71] Angley LP Combs M Firth C Frye MJ Lipkin I Richardson JL Spatial variation in the parasite communities and genomic structure of urban rats in New York City. Zoonoses Public Health. 2018;65(1):e113-23. 10.1111/zph.12418 29143489

[72] Byers KA Booker TR Combs M Himsworth CG Munshi-South J Patrick DM Using genetic relatedness to understand heterogeneous distributions of urban rat-associated pathogens. Evol Appl. 2020;14(1):198-209. 10.1111/eva.13049 33519965 PMC7819557

[73] Richardson JL Silveira G Soto Medrano I Arietta AZ Mariani C Pertile AC Significant genetic impacts accompany an urban rat control campaign in Salvador, Brazil. Front Ecol Evol. 2019;7:115. 10.3389/fevo.2019.00115

[74] Davis DE . The characteristics of rat populations. Q Rev Biol. 1953;28(4):373-401. 10.1086/399860 13121239

[75] Nygren NV Tuomas JA . Rotta kuntalaisena – rottien esiintyminen ja hallinta. [Rat as a municipal citizen – rat occurrence and control]. Suom Eläinlääkl. 2022;128(6):331-7.

[76] Lee MJ Byers KA Donovan CM Bidulka JJ Stephen C Patrick DM Effects of culling on Leptospira interrogans carriage by rats. Emerg Infect Dis. 2018;24(2):356-60. 10.3201/eid2402.171371 29350160 PMC5782904

[77] Bagan H Yamagata Y . Land-cover change analysis in 50 global cities by using a combination of Landsat data and analysis of grid cells. Environ Res Lett. 2014;9(6):064015. 10.1088/1748-9326/9/6/064015

[78] Nygård H. Kompostoida vaiko polttaa? keskustelu jätteenkäsittelyn vaihtoehdoista 1950-luvulla. In: Näkökulmia Helsingin ympäristöhistoriaan [Compost or incinerate? discussion of alternatives to waste treatment in the 1950s. In: Perspectives on Helsinki's environmental history]. Helsinki: Edita/Helsingin kaupungin tietokeskus; 2001. p. 90-101.

[79] Sane J Ollgren J Makary P Vapalahti O Kuusi M Lyytikäinen O . Regional differences in long-term cycles and seasonality of Puumala virus infections, Finland, 1995-2014. Epidemiol Infect. 2016;144(13):2883-8. 10.1017/S0950268816000765 27113030 PMC9150413

[80] Stryjek R Kalinowski A Parsons MH . Unbiased sampling for rodents and other small mammals: How to overcome neophobia through use of an electronic-triggered live trap-A preliminary test. Front Ecol Evol. 2019;7(FEB):11. 10.3389/fevo.2019.00011

[81] Robinson SJ Pearl DL Himsworth CG Weese JS Lindsay LR Dibernardo A Environmental and sociodemographic factors associated with zoonotic pathogen occurrence in Norway rats (Rattus norvegicus) from Windsor, Ontario. Zoonoses Public Health. 2024;71(4):416-28. 10.1111/zph.13120 38419369

